# Wake up call from ‘Stars on the Ground’

**DOI:** 10.4103/0019-5545.39749

**Published:** 2008

**Authors:** T. S. Sathyanarayana Rao, V. S. T Krishna

**Affiliations:** Department of Psychiatry, JSS Medical College Hospital, Mysore - 570 004, India

It might not be over stating, to say that movies have made inroads within the minds of the public since their inception. The Indian subcontinent too, is in line with this global scenario. Perhaps it will be a hard task to find a matching medium on par with movies in terms of the magnitude of the impact on the public psyche. Movies have immense potential in depicting the intricacies of human life. Further, movies go a long way in touching human lives in great detail. Unlike any of the other medium which humans have invented so far, movies are influencing persons hailing from all cross sections in our society, irrespective of their age, sex, education, occupation, intellectual levels and socio-economic and cultural backgrounds. These comments are of more relevance while one ponders over a recent Indian movie, “Taare Zameen Par” (Stars on the Ground), with a subtitle “Every child is special” as a timely reminder of an oft forgotten truth.

The impact of the medium was borne out once again to us. A 19 years moderately retarded young boy reported with a series of complaints as stated by the relatives: that he would get up in the middle of the night and wander at large, to return in the morning hours and sleep in the daytime. During his roaming in the night hours it was reported that he gate- crashed into neighboring households and stole the dresses of women. Further, he behaved excitedly while interacting with women and tried to pull their hair and tug at their sarees and he would burst into laughter at his own acts. It was also reported that he refused to eat food, becoming aggressive and shouting at family members and was resorting to disrobing himself on the roads, often dancing nude.

Unable to bear his wild acts and huge neighbourhood pressure, his family members had admitted him to a government psychiatric facility and he was kept on anti-psychotics and ECTs. The outcome was very poor. Following this, he was admitted into a rehabilitation facility. After repeated home visits by a psychiatric social worker, this young boy was encouraged to participate in a supportive social group interaction. In this social group in spite of his compromised communication skills, this boy opened up to express his experiences and feelings with considerable clarity. He revealed that he had been trying to imitate the heroes of films! Further, he stated that he felt dejected and appalled at being beaten up whereas film heroes are envied and applauded for the same kind of deeds on celluloid.

Following this revelation the treatment process and the approach to this patient was drastically changed and the boy started showing good progress. A key aspect of this case study to be noticed seriously is the stupendous power of films and their ability to affect the thought process, as well as the emotional and behavior repertoire of different sections of our society. The recent gun-shootings in the school corridors in India are also to be reviewed from the dimension of the role of media, especially the films and their impact on school children.

On the other side of the coin there are several movies which can be cited which have demonstrated positive effects on the psyche too. There is little doubt that Mr. Shahrukh Khan could ignite team spirit and drive to fight out odds, through his “Chak de India” performance. The passionate patriotism was in line with Aamir Khan's classic film “Lagaan”. Even “Swadesh” could move many well qualified technocrats across the globe to feel for India. By the clarion call given by the role of space scientist, played by Mr. Shahrukh Khan sensitized viewers very earnestly to feel for one's own motherland, not to forget the smell of the soil where one is born and brought up, to respect our roots and to bond with the people of the land

A keen psychiatrist, or for that matter any other concerned mental health professional can not remain aloof from the fact that movies are doing a plausible job in grasping the challenges to mental health issues and sometimes offering creative remedies. Now the time is right to get inspired (and then transmit such inspiration) generated by the valiant endeavors like Mr. Aamir Khan's “Taare Zameen Par”, a film clearly out of the ordinary. This movie stands distinguished not just as being highly socially conscious but it wakens us to important tasks before this nation and all concerned mental health professionals, agencies, policy makers and not the least educationists at various levels, as well as administrators, “Taare Zameen Par” deserves to be vastly appreciated as an earnest endeavor to portray with sensitivity and empathetically diagnose a malady in human life. A young child fights valiantly, a stern social and emotional environment; as he gets locked in an unsuccessful fight for grades, his family and school turn increasingly hostile. The hapless and helpless parents transfer him to a boarding school expecting its strict regimen to help him shape up. It takes a teacher with rare sensitivity in this new school to recognize the protagonist's learning disorder. Further, this picture presents an optimistic approach, in the process of troubleshooting endeavor by blending modern professional knowledge coupled with a humane approach in working with a child having a disability such as dyslexia.

It will be acutely underestimating the content of this film if we narrow it down merely as an exercise in recognizing and managing dyslexia. In fact, it looks like a wide angle landscape of the present age where everyone is in a restless hurry. This film raises serious questions on mental health perspectives. We seem to be heading to a state of mass scale mindlessness even as children are being pushed to ‘perform’. Are we seriously getting engrossed in the race of “achievement” and blissfully becoming numb to the crux of life i.e., experiencing meaningful living in a broader frame rather than merely existing?

Further, this feature film raises a serious alarm subtly asking us to note the impending danger of collapsing standards of mental health: family systems hell bent on goading young children to peaks of achievement, and schooling systems governed by steel frames of critical disciplinary forces. Are we heading to a major land slide in the coming years leading to major breakdowns in the mental health and functioning of school going children? Having heard recently the gunshots in our school corridors, what are we waiting for? We need to wake up to the serious realities that the film depicts and adresses many avoidable ills. The responsibility is all the more onerous for all mental health workers.

Let us allow the blossoming of laughter and nurture the brimming, bubbling enthusiasm of childhood in the classrooms and corridors of schools. Gun shots in school campuses and as well the “Tare Zameen Par” protagonist, Ishan Awasthi's break down are not mere misfortunes. They are life size mirrors of failure existing in society and the same society's failure to rise to the need of the hour. Even as we write this editorial, another issue related to the country's crazy preoccupation with academic achievement is being brought into focus through yet another malady: adolescents committing suicide in the face of milestone examinations or unable to face their results.

In the name of raising deafening noise of progress, let us not turn deaf to the voices of children's needs; let there not be any massacre of sensitive childhood dreams. It is a national responsibility to ensure that childhood shall not become an endangered period of human life, lest we need to be ready to experience ghastly human tragedies and maladies in times to come and that day may not be far if we continued to ignore the challenging issue of providing School Mental Health Services. One needs to seriously probe the fact that positive mental health promotion is not a cross sectional element, rather in reality, it is a longitudinal journey starting from early childhood.

We should perhaps be grateful that we as yet have not devised programmes to force academics on infants! How justified we are in punishing a 2 or 3 year old child for not learning alphabets in the correct order? Are we entering into a world of maddened by the need for academic achievement? Ishan Awasthi cornered rudely for a poor school performance without probing into his strengths and weaknesses, runs away from the maddening crowd, as well might millions of our children. This film vividly portrays the valiant efforts of Ishan Awasthi – played immaculately by Darsheel Safary- in the role of a child with learning disability. We can visualize a symbolic representation of the predicaments of schooling being faced by an average child, ultimately representing the urban national scenario too.

It is not far from truth or in any way antisocial if Ishan Awasthi tries to put a forged “absent note” after he escapes school in an attempt to cope with his inner struggle, further spurred by a cruel school system. If this withdrawal strategy is limited to a mere psychiatric diagnostic perspective, then we may be underestimating the potential ‘tsunami’ in the form of a social disorganization which would knock down large numbers of school goers within a short span of time. Both the family system and the school do have their tremendous contributions towards this end.

Are we getting excessively Darwinian by heading towards “Survival only for the fittest?” If this remains the state of affairs, what would be the role of helping professions and mental health advocates? The picture portrays that strategies that only use threats and coercion are not capable of unearthing rich human potentialities deeply embedded in children. Rather, it is the need of the hour to skillfully and sensitively gear up the children to the required levels of performance by carefully mapping their strengths, weakness and resources. Further, this film has made an attempt of recognizing that each child is special in his own way with unique needs, drives, weaknesses and above all distinguishing potentialities. “Taare Zameen Par” boldly speaks and loudly says that sparing the rod shall be the order of the day and caring the child is the need of the hour. Aamir Khan dexterously drives home the precise point that our first priority ought to be getting to know the child before making any efforts to fill them with knowledge and abilities. Aggression does not pay in instilling education, so blunt punishments have almost no role to play.

The heart of the film is to pass the message to the viewer that the skill of empowering a below average performer needs on first priority the sensitive efforts of relating, relating and relating alone; for this above all works with the child. Only then can one move him upward the ladder. But unfortunately relating is displaced or replaced by coercion and drudgery: piles of homework, conveyor belt assignments and unrealistic deadlines. Unless you relate you can't locate a child's deficiencies and unless this is done, there is no scope for filling the gaps. This is the punch line of this film.

Ram Shankar Nikumbh, the perceptive teacher portrayed sensitively by Aamir Khan, politely tries to remedy his colleague teachers, delicately reminding them that they have forgotten how to laugh heartily and that they are driven only by a “performance engine'. He impresses upon them that there is no point in pressurizing children to perform all the way; rather it is beneficial to develop their innate urge to perform. Thus he shows the road to excellence. He tries to transform them to states of flexibility and makes them receptive to variations in the creative needs of children and thus to facilitate more amenability. He does his best to ensure that they become more communicative to their students by engaging them in creative arts and painting where there is emphasis on expression and respect for every form of expression.

If there is a fault in ‘Taare Zameen Par's content, it is in its naïve oversimplification. In a country only recently waking to recognizing the reality and tragedy of learning disability, this is easily forgivable under artistic license.

Are we ready to take the message seriously? For having crystallized several sensitive issues concerning child development, in a feature film form, let us say Kudos to Aamir Khan and his team.

